# Vertical platysma myocutaneous flap that sacrifices the facial artery and vein

**DOI:** 10.1186/1477-7819-11-165

**Published:** 2013-07-24

**Authors:** Zhen-ning Li, Rui-wu Li, Fa-yu Liu, Qi-gen Fang, Xu Zhang, Chang-fu Sun

**Affiliations:** 1 Department of Oromaxillofacial-Head and Neck Surgery, China Medical University, 117 South Nanjing Street, Heping District, Shenyang 110002, China; 2 Department of Oral and Maxillofacial Surgery, School of Stomatology, China Medical University, 117 South Nanjing Street, Heping District, Shenyang 110002, China

**Keywords:** Vertical platysma myocutaneous flap, Facial artery and vein, Intraoral reconstruction

## Abstract

**Background:**

Platysma myocutaneous flap (PMF) is a generally used technique for defect reconstruction after an oral cancer resection. The aim of the study is to present our experience using vertical PMF that sacrificed the facial artery and vein for intraoral reconstruction.

**Methods:**

A retrospective review of the medical records of 54 patients who underwent vertical PMF that sacrificed the facial artery and vein for intraoral reconstruction was performed. A comparison between PMF that sacrificed and that preserved the facial vessels was made, and we also compared PMF that sacrificed the facial vessels with radial forearm free flap (RFFF). Statistics concerning the patients’ clinical factors were gathered.

**Results:**

The mean age of the 54 patients who underwent PMF that sacrificed the facial artery and vein was 62.0 ± 10.98 years. The co-morbid disease rate of PMF was 53.7%. The flap size ranged from 12 × 5.5 cm to 7 × 5 cm. Survival of the flap was found in all of the cases, with partial necrosis in four cases (7.4%) and total loss in none of the cases. The operation time was 5.7 ± 1.17 h. The complication and success rates were 27.8% and 92.6%, respectively. The 3-year and 5-year survival rates were 77.8% (21/27) and 69.23% (9/13), respectively. The majority of the patients (87.0%) in our series were satisfied with the results of the surgery. There was no significant difference between PMF that sacrificed or that preserved the facial vessels, both in success rate (*P* = 1) or complication rate (*P* = 0.72). The patients in the PMF group were older than the patients in the RFFF group (*P* = 0.011), the operation time was shorter (*P* < 0.001), and the co-morbid disease rate was higher (*P* = 0.002). Although the complication rate of PMF (15/54, 27.8%) was higher than that of RFFF (2/34, 5.9%) (*P* = 0.011), their success rates were similar (92.6%, 94.1%) (*P* = 1.00).

**Conclusions:**

Vertical PMF that sacrifices the facial artery and vein has specific advantages including in ease preparation and limitations. This technique may provide an effective method for intraoral reconstruction. Our experience in handling the flap may contribute to the success rate.

## Background

To repair a defect after intraoral cancer excision, several factors should be considered, such as the site and complexity of the defect, the expertise of the surgeon, and the need for coverage of the great vessels [1]. Although numerous options can be used for reconstruction of the head and neck, including primary closure, skin grafts, pedicle flaps, healing by secondary intentions, and free flaps, all of these techniques have their limitations [2]. With the development of microsurgical techniques in recent years, free flaps, such as radial forearm flaps, latissimus dorsi flaps, and anterolateral thigh free flaps, have a tendency to predominate intraoral reconstruction. However, free flaps are not suitable for all patients undergoing a surgical operation. The reconstruction plan should be determined according to the patient’s general body state, potential complications, socioeconomic status, color, texture, and skin characteristics of the flap with respect to the recipient region, whether two operational sites are needed and the surgeon’s microsurgical experience.

Platysma myocutaneous flap (PMF) is a satisfactory reconstructive option for small- and medium-sized defects in the oral cavity. This technique was first introduced in the literature for intraoral reconstruction in 1978 [3]. Since then, PMF has been generally used for the reconstruction of congenital abnormalities, traumatic injuries, and, most commonly, malignancies of the head and neck [4]. Three types of PMF have been described: transverse flap, vertical flap that preserves the facial artery and vein, and vertical flap that sacrifices the facial artery and vein. Some authors have reported that vertical PMF without the preservation of the facial vessels is unreliable [5].

In this article, we demonstrate an interesting result in patients who underwent vertical PMF that sacrificed the facial artery and vein for intraoral reconstruction.

## Methods

### Patients

We retrospectively reviewed the medical records of 54 patients who underwent vertical PMF reconstruction of an intraoral defect after a primary tumor excision from January 2005 to September 2012 at the Department of Oral and Maxillofacial Head and Neck Surgery, Department of Oral and Maxillofacial Surgery, School of Stomatology, China Medical University. Modified radical or selective neck dissection was also performed on the patients. All 54 cases were reconstructed with vertical flaps that sacrificed the facial artery and vein. Tumor excision and defect reconstruction were both performed by the same team. The age and sex of the patients, location and size of the lesions, surgical complications, and outcome were recorded. We obtained the histological diagnoses from the resected specimens. Postoperative death was defined as death before being discharged from the hospital. A comparison between PMF that sacrificed the facial vessels and PMF that preserved the facial vessels was made, and we also compared PMF that sacrificed the facial vessels with radial forearm free flap (RFFF). Informed consent, including one item that patients are willing to dedicate all the medical data (photos, medical records, specimens, etc.) to medical research and academic exchanges, was obtained from all of the patients involved in the research. And approval was obtained from the ethics committee of School of Stomatology, China Medical University.

### Surgical technique

**Figure 1:**
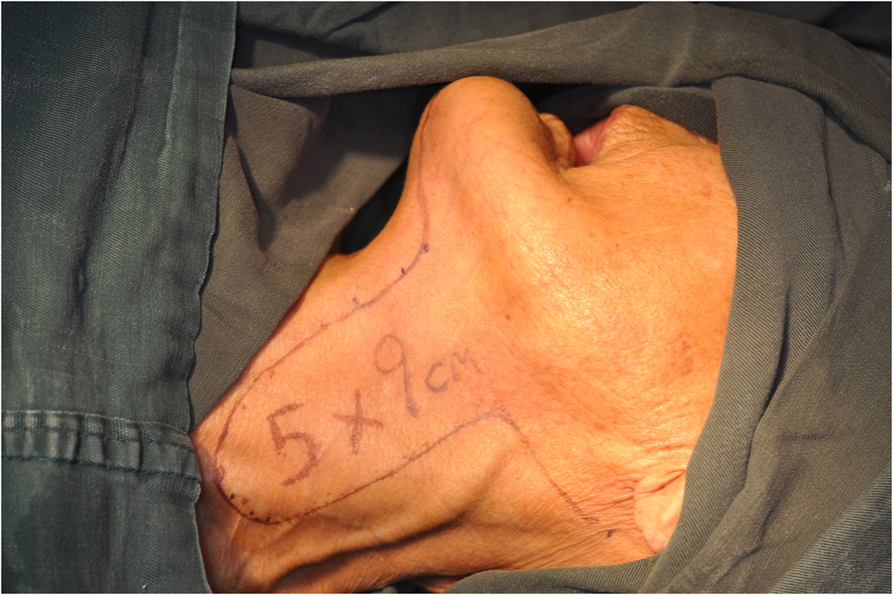
Design of the platysma myocutaneous flap.

**Figure 2:**
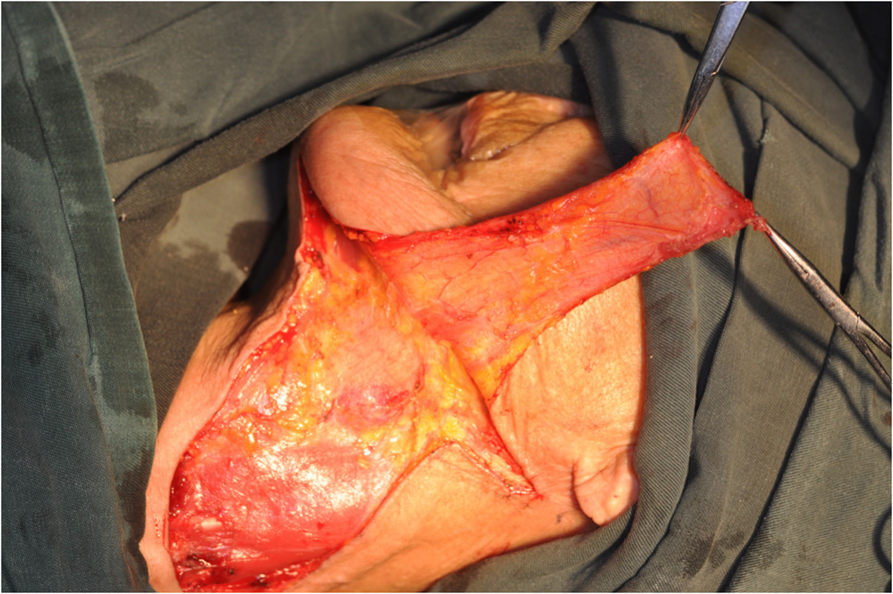
The flap was harvested using sharp dissection with a surgical knife under the adipofascial tissue below the platysmal plane.

**Figure 3:**
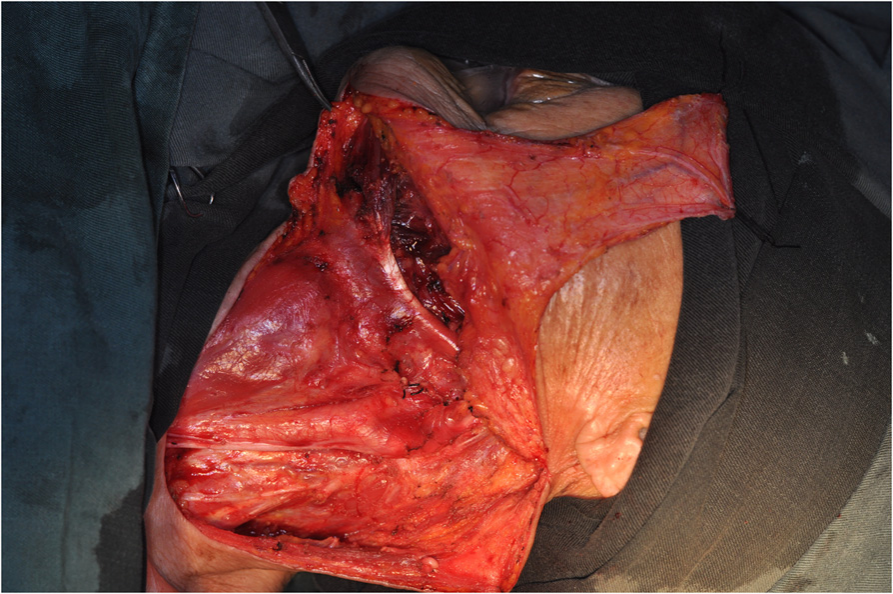
Neck dissection was completed with the facial artery and vein ligated.

**Figure 4:**
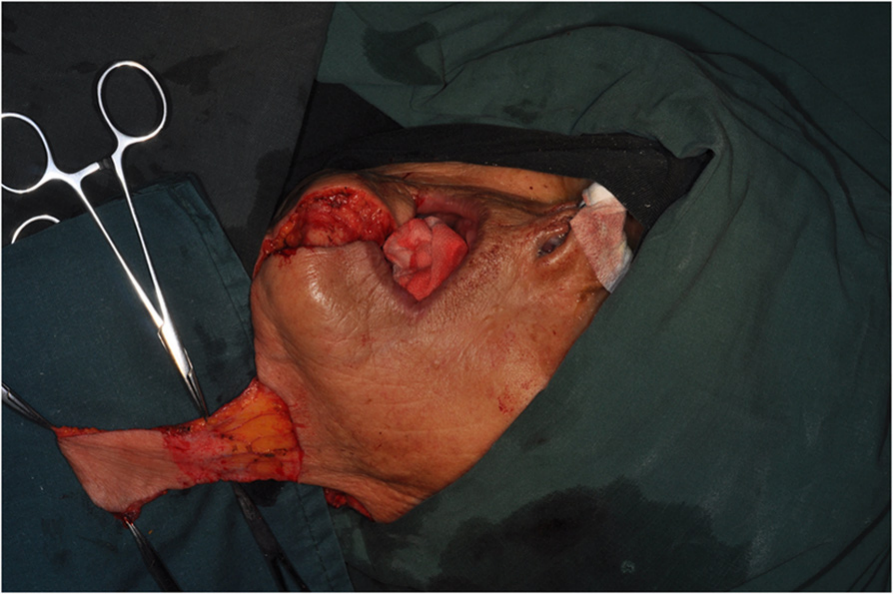
Before reconstructing the defect, the epidermis was removed from part of the flap in the tunnel.

The size of the flap was designed according to the anticipated defect resulting from the excision of the primary tumor (Figure 1). Vertical PMF was prepared and raised in the ipsilateral neck of the defect to be repaired, and the incision line was often connected with that of the neck dissection. The flap pedicle was usually located 1.5 cm to 2 cm below the lower border of the mandible. The aspect ratio of the pedicle was mostly 2:1. The flap was harvested using sharp dissection with a surgical knife under the adipofascial tissue below the platysmal plane (Figure 2). Then, the neck dissection and tumor resection were performed. The facial artery and vein were all ligated in these cases (Figure 3). The pedicle should not be compressed or turned around before rotating and transporting it through a tunnel, which should be sufficiently wide. The epidermis was removed from the part of the flap located in the tunnel (Figure 4). Due to the sufficient length, excessive stretching and suture tension on the flap were avoided. When closing the incision in layers, two suction catheters should be placed for postoperative drainage.

### Statistical analysis

The Student’s t-test and the χ2 test were used to study the statistical difference of the variables. A *P* value <0.05 was considered significant. The evaluation of postoperative function was performed according to the method described by Hell *et al.*[6]. A score of 7 points was rated as excellent, 6 and 5 points as good, 4 points as fair, and <4 points was considered poor. For this reason, a patient with a score >5 points was considered to show good restoration of tissue function.

## Results

In our series, 54 patients received vertical PMF that sacrificed the facial artery and vein reconstruction after receiving treatment for an intraoral carcinoma. The medical records of these patients were reviewed before surgery. One of the patients had undergone preoperative radiotherapy. Forty patients were men, and the male/female ratio was 2.9/1. The patients were aged between 38 and 80 years (mean, 62.0 ± 10.98 years). The span of operation time ranged from 2.50 h to 9.00 h (mean, 5.7 ± 1.17 h). The stages of cancer reported for these patients ranged from T1N0M0 to T4N2bM0 (Table 1). After tumor excision, the size of the defects ranged from 3.5 × 3 cm to 6.5 × 5 cm. For the vertical flaps, the maximum size was 12 × 5.5 cm and the minimum was 7 × 5 cm. Most of the defects that were reconstructed involved the tongue (32.2%) and the floor of the mouth (37.3%). Final survival of the vertical PMF that sacrificed the facial artery and vein was found in all of the cases, with epidermal loss in 11 cases (20.4%) and partial necrosis in four cases (7.4%); total loss did not occur in our patients. The buccal mucosa (4.4%) and tongue (43.5%) were the recipient sites with the lowest and the highest risk of postoperative complications, respectively (Table 2). Four patients developed multiple complications. In the 50 cases with postoperative complications, six cases had co-morbid disease, while in the 29 cases with co-morbid disease, six cases had postoperative complications. No relationship between the complications and co-morbid disease was found (*P* = 0.21). Eventually, fibrosis and gradual recovery was observed in the four patients with partial necrosis. Twelve patients (44.4%) received postoperative radiation 3 weeks after being discharged from the hospital, and five patients experienced partial excoriation, which disappeared completely 4 to 6 weeks after radiation therapy. No subsequent flap failure or necrosis due to the side effects of radiation was observed. No postoperative death was observed, and there were no serious voice difficulties or limitations in mouth opening in any of the patients. No patients experienced any restriction in neck movement. All of the patients with basic oral cavity function and acceptable neck scars experienced complete wound healing 6 months after the operation. The follow-up continued until September 2012. In our study, 27 patients participated in regular follow-ups, but the other patients did not follow up after being discharged from the hospital. The mean follow-up duration was 4.2 ± 1.92 years, ranging from 1 to 8 years. Twelve patients (44.4%) died from their disease due to local recurrence. The 3-year and 5-year survival rates were 77.8% (21/27) and 69.23% (9/13), respectively. The majority of the patients (87.0%) in our series were satisfied with the results of the surgery.

**Table 1:** Patients and tumor characteristics

**Characteristics**	**Patients ( *n *,%)**
*Sex*	
Male	40 (74.1)
Female	14 (25.9)
Sex Ratio (male/female)	2.86
*Age (years)*	
Median	62 ± 10.98
Range	38-80
*Preoperative radiation therapy*	1 (1.9)
*Postoperative radiation therapy*	12 (44.4)
*Site*	
Buccal mucosa	9 (15.3)
Tongue	19 (32.2)
Alveolar ridge	6 (10.2)
Floor of mouth	22 (37.3)
Pharyngeal wall	2 (3.4)
Soft palate	1 (1.7)
*T stage*	
T1-T2	34 (63.0)
T3	10 (18.5)
T4	10 (18.5)
*Total patients*	54 (100.0)
*N stage*	
N0	44 (81.5)
N1	4 (7.4)
N2a	3 (5.6)
N2b	3 (5.6)
*Total patients*	54 (100.0)

**Table 2:** Complication rates of different recipient sites

**Recipient site**	**Partial necrosis (%)**	**Epidermis loss (%)**	**Fistula (%)**	**Infection (%)**	**Patients ( *n *,%)**
Floor of mouth	2 (28.6)	3 (42.9)	1 (14.3)	1 (14.3)	7 (30.4)
Alveolar ridge	0 (0.0)	1 (50.0)	0 (0.0)	1 (50.0)	2 (8.7)
Tongue	2 (20.0)	5 (50.0)	1 (10.0)	2 (20.0)	10 (43.5)
Pharyngeal wall	0 (0.0)	1 (33.3)	1 (33.3)	1 (33.3)	3 (13.0)
Buccal mucosa	0 (0.0)	1 (100.0)	0 (0.0)	0 (0.0)	1 (4.4)
All sites	4 (17.4)	11 (47.8)	3 (13.0)	5 (21.7)	23 (100.0)

There were 35 patients with one or more complications.

Of the 29 patients whose facial vessels were preserved, two cases had partial necrosis, five cases had epidermal loss, and no cases had total loss. The complication and success rates were 24.1% and 93.1%, respectively. There was no significant difference between PMF that sacrificed or preserved the facial vessels, both in success rate (*P* = 1.00) or complication rate (*P* = 0.72) (Table 3).

**Table 3:** The comparison of PMF sacrificed or preserved the facial vessels

**Flap types**	**Total ( *n *)**	**Successful No.**	**Complications**			**Success rate (%)**
			**TL**	**PN**	**EL**	
PMF sacrificed the facial vessels	54	50	0	4 (7.4%)	11 (20.4%)	92.6
PMF preserved the facial vessels	29	27	0	2 (6.9%)	5 (17.2%)	93.1

TN: total loss. PN: partial necrosis. EL: epidermis loss.

The operation time for patients in the PMF group was 5.7 ± 1.17 h, and the operation time was 8.4 ± 2.15 h for the RFFF group (*P* < 0.001). The mean age of patients in the PMF group was 62.0 ± 10.98 years, and the mean age was 55.6 ± 11.44 years in the RFFF group (*P* = 0.011). Co-morbid disease was observed in 10 cases (10/34, 28.6%) in the RFFF group, which was lower than the PMF group (*P* = 0.002). Total loss occurred in two cases (2/34, 5.9%), and the success rate was 94.1% (Table 4).

**Table 4:** The comparison between PMF sacrificed facial vessels and RFFF

**Items**	**PMF**	**RFFF**
Age (years)	62.0 ± 10.98	55.6 ± 11.44
Co-morbid disease rate	53.7% (29/54)	28.6% (10/34)
Operation time (h)	5.7 ± 1.17	8.4 ± 2.15
Complication rate	27.8% (15/54)	5.9% (2/34)
Success rate (%)	92.6	94.1

The operation time include the time of unilateral neck dissection in both groups.

## Discussion

Malthes and Nahai described the ‘Reconstructive Ladder’ in 1982, which is based on the complexity of surgery, and they explained that reconstruction should be performed with simple and complex techniques, ranging from primary closures to skin grafts, local flaps, distal flaps, and free flaps [7]. Due to the flap’s thinness, location, pliability, ease of preparation, and number of limitations as well as the lack of hair found in the patients of Asian descent, PMF has been widely used for the reconstruction of head and neck defects, including the ear, cheek, upper and lower lips, oral cavity, and oropharynx [8-12]. The platysma receives most of its blood supply through the submental artery, which is the largest branch of the facial artery, and this muscle also receives blood due to anastomosis of the ipsilateral and contralateral lingual, superior thyroid and inferior labial arteries [13]. A recent anatomical investigation revealed the inherently fasciocutaneous nature of the platysma flap [14]. When the facial artery is ligated, there is retrograde filling of the platysma system through both ipsilateral internal and external carotid contributions as well as contralateral external carotid contributions through the labial arch [15]. Some studies have suggested that the contraindications of using PMF include ligation of the facial artery, previous neck surgery, radiotherapy, ipsilateral facial nerve paralysis, and prior radical neck dissection [16-18]. Su *et al.* reported in his study that in 16 cases of vertical PMF that sacrificed the facial vessels, four cases suffered from partial necrosis and two had total loss and that, in 26 cases of vertical PMF that preserved the facial vessels, three cases suffered from partial necrosis and none had total loss [5]. The success rates of the two flaps were 62.5% and 88.5%. Table 3 shows the comparison between PMF that sacrificed the facial vessels and PMF that preserved the facial vessels in our study. In our patients with sacrificed facial vessels, we found that 50 (92.6%) cases survived, four cases (7.4%) had partial necrosis and none had total loss. Of 29 patients with preserved facial vessels, two cases had partial necrosis, five cases showed epidermal loss, and none had total loss. Finally, the success rates of the two flaps were 92.6% and 93.1%. There were no significant differences between the two groups, both in success rate (*P* = 1) and complication rate (*P* = 0.72). Based on our data, PMF that sacrifices the facial vessels may have a reliability similar to that of PMF that preserves the facial vessels. However, Berenholz *et al.* believe that the facial artery and vein should not be preserved when there is a submandibular lymph node metastasis, and the use of the platysma flap should be avoided [19]. Therefore, we believe that the indication of PMF that sacrifices the facial vessels should be more extensive.

We believe that the techniques used during the operations may have a high success rate due to these reasons:

1. To preserve the anastomosing vascular network between the platysma and the fascia, the deep adipofascial tissue under the platysma was carefully protected when harvesting the flap.

2. Although the external jugular veins were ligated, they should be elevated with the flap if possible. We believe that the remaining veins may contribute to the reestablishment of blood circulation.

3. To protect the blood supply, only the epidermis, instead of the full thickness of the skin, is removed from the surface from the pedicle of the flap.

4. Concerning the complete resection of the tumor, excessive removal of the surrounding tissue, especially the distal subcutaneous tissue, should be avoided. Subsequently, the distal region of the flap may receive an improved blood supply from the peripheral neovascularization between the flap and its bed.

5. When transporting the flap into the oral cavity, excessive torsion, stretching, and suture tension should be avoided. The tunnel for the flap should also be expanded properly.

6. When closing the incision on the donor site of the neck, the suture over the pedicle, below the lower border of the mandible, should be made in the epidermis and superficial dermis in a vertical direction. We believe that this practice would contribute to preservation of the blood supply.

Additionally, tumor excision and defect reconstruction are performed by the same team in our hospital; thus, the surgeons may have a better understanding of the specific circumstances of the operation. This experience may lead to a greater chance of flap survival.

Currently, radial forearm free flap (RFFF), with the advantages of reliable anatomy, long pedicle length, appropriately-sized vessels, and suitable thinness, is widely used in reconstructive head and neck surgery [20]. Due to similarities between the two techniques, we compared the vertical PMF that sacrificed the facial vessels group and the RFFF group after intraoral defect reconstruction at the same period in our hospital (Table 4). We found that the operation time for patients in the PMF group (mean, 5.7 ± 1.17 h) was shorter than the RFFF group (mean, 8.5 ± 2.16 h), which was significantly different (*P* <0.001), indicating that PMF was faster and simpler to perform than RFFF. The co-morbid disease rate (53.7%) of PMF was much higher than that of the RFFF group (28.6%) (*P* = 0.002), which indicated that PMF was more suitable for patients with a poor general body state. The mean age of patients in the PMF group was 62.0 ± 10.98 years, which was almost 7 years older than the RFFF group (mean, 55.6 ± 11.44 years) (*P* = 0.011), which indicated that PMF was more suitable for older patients. Of the 54 PMF performed, partial necrosis occurred in four cases (4/54, 7.41%), and total loss did not occur. Total loss occurred in two cases (2/34, 5.9%) in the RFFF group. Although the complication rate of PMF (15/54, 27.8%) was higher than that of RFFF (2/34, 5.9%) (*P* = 0.011), their success rates were similar (92.6%, 94.1%) (*P* = 1.00). We believe that the reason for the high success rate is because in PMF, the tunnel can fill the tissue volume defect between the surface and deeper tissues, rather than only cover the wound. If necrosis occurs in the distal region of the flap, the maintained, underlying viable muscles can promote rapid re-epithelialization. As a result, final survival of the flap can also be achieved, rather than total loss. The impact of the complications in the PMF group was lower than that of RFFF, indicating that PMF might be safer than RFFF. Furthermore, we also believe that the low complication rate of the free flaps may have a relationship with the strict indications. Based on the data above, we believe that PMF, to some degree, may have more extensive indications than RFFF.

Nevertheless, a disadvantage of vertical PMF is its insufficient venous drainage [21]. The variability in the length of the patient’s neck and the thickness of the platysma may also be factors that influence the use of the flap. Preoperative radiation was reported as a contraindication for the use of PMF [17]. However, Szudek *et al.*[1] believe that postoperative complications were not associated with preoperative radiation therapy. In our study, there was one patient who received preoperative radiation therapy and showed good restitution. Further research is required to determine whether preoperative radiation therapy affects the flap.

## Conclusions

Vertical platysma myocutaneous flap that sacrificed the facial artery, with the specific advantages of being easy to prepare and having few limitations, may provide an efficient method for intraoral reconstruction, and our experience in handling the flap may contribute to the success rate.

## Competing interests

The authors declare that they have no competing interests with any other person or organization.

## Authors’ contributions

ZL, FL, and CS wrote the manuscript. QF and XZ performed the statistical analysis of the studies. CS and RL participated in the design and performance of the operations. All of the authors have read and approved the final manuscript.
